# Screening of potential tropical fruits in protecting endothelial dysfunction *in vitro*

**DOI:** 10.29219/fnr.v65.7807

**Published:** 2021-09-01

**Authors:** Suvara K. Wattanapitayakul, Khwandow Kunchana, Wattanased Jarisarapurin, Linda Chularojmontri

**Affiliations:** 1 Department of Pharmacology, Faculty of Medicine, Srinakharinwirot University, Bangkok, Thailand; 2 Department of Preclinical Sciences, Faculty of Medicine, Thammasat University, Bangkok, Thailand

**Keywords:** endothelial cells, nitric oxide, functional food, nutraceuticals, cardiovascular diseases

## Abstract

**Background:**

High consumption of antioxidant-rich fruits and vegetables reduces the endothelial damage involved in cardiovascular disease pathogenesis.

**Objective:**

To evaluate the phytochemical content, antioxidant and scavenging activities (FRAP, ORAC, OH^•^, HOCl, H_2_O_2_, and O_2_^−^), endothelial H_2_O_2_-cytoprotective effect, nitric oxide (NO) release activation potential, and endothelial wound healing properties of 10 tropical fruits, comprising pineapple, sugar apple, papaya fruit, longan, mangosteen, lychee, langsat, mango, rambutan, and guava.

**Design:**

Experimental study. The experiments were conducted *in vitro* using endothelial cell line EA.hy926.

**Results:**

The high performance liquid chromatography (HPLC) phytochemical analysis indicated the presence of gallic acid and quercetin in all fruits, along with the overall absence of ellagic acid. Chlorogenic acid was only detected in three fruits, that is, pineapple, ripe papaya, and guava. The antioxidant and scavenging activities of all fruits were concentration-dependent. Only the H_2_O_2_ scavenging activity exhibited broad positive associations with other ROS-scavenging activities. Sugar apple and unripe papaya induced a significant reduction in H_2_O_2_-induced cell death in endothelial cells while pineapple, sugar apple, longan, and langsat activated NO release.

**Discussion:**

All the studied tropical fruits contained bioactive phytoantioxidants with wide ranges of antioxidant capacity and scavenging activities. The endothelial functional tests were relevant to the screening for fruits that may benefit cardiovascular health. Among the four fruits that promoted endothelial wound closure, only sugar apple and unripe papaya induced cell migration and vascular capillary-like tube formation.

**Conclusion:**

Sugar apple and unripe papaya are potential functional fruits that can protect against oxidative cell death and enhance endothelial wound healing.

## Popular scientific summary

- Fruits and vegetables contain bioactive antioxidants that can lower risks of stroke and coronary heart disease by protecting endothelial cells from oxidative damage and dysfunction.
- Among 10 selected tropical fruits, sugar apple and unripe papaya protect endothelial cell injury and maximize endothelial wound repair while pineapple, sugar apple, longan, and langsat promote endothelial function.
- These functional fruits can play a significant role in dietary recommendations to reduce the risks of stroke and cardiovascular disease.

Reactive oxygen species (ROS) at well-balanced levels function as signaling molecules and are required for physiological cellular activities. However, excessive ROS generation, termed ‘oxidative stress’, contributes to metabolic and cardiovascular diseases (CVDs) (1). The endothelial layer, lining the lumen of blood vessels, is crucial for maintaining vascular homeostasis and controlling blood pressure (2). A key function of endothelial cells is nitric oxide (NO) release, serving as a vascular relaxant, immune cell adherence regulator, and platelet aggregation inhibitor. Endothelial function impairment is a well-recognized early event in CVD pathogenesis (3). The reduction in NO production and endothelial cell damage are the typical causes of endothelial dysfunction, leading to hypertension, atherogenesis, or wound healing impediment (4, 5). Long-term accumulation or ROS overproduction, especially long-term accumulation of hydrogen peroxide, superoxide, and hydroxyl radical, is reportedly related to endothelial dysfunction and apoptosis (6). Thus, the therapeutic targeting of ROS scavenging with antioxidants is a heavily studied recent field of research.

The naturally-occurring cellular antioxidant defense, essential for maintaining the ROS level balance, could be broadly divided into two categories, including enzymatic and non-enzymatic antioxidants. The enzymatic antioxidant system, specifically protecting cells from oxidative damage, is induced by hydrogen peroxide (catalase, CAT, and glutathione peroxidase, GPx), superoxide (superoxide dismutase, SOD), while the hydroxyl radical could be eliminated by small-molecule antioxidants. The non-enzymatic antioxidants are present both endogenously, such as in the form of glutathione, coenzyme Q, and alpha-lipoic acid, and as plant-derived vitamins and phytoantioxidants, such as vitamin C and E, flavonoids, polyphenols, or tannoids. Recognizing the benefits of antioxidants, the consumption of natural and synthetic antioxidants as dietary supplements is on the rise (7). Nonetheless, the major concerns in CVD prevention using synthetic antioxidant vitamins were stimulated by the meta-analyses, showing unsatisfactory results for the disease risks or mortality reduction attempts (8, 9). In contrast, the regular and high-dose consumption of fruit and vegetable (F&V)-derived dietary natural antioxidants correlates well with disease risk reduction with emphasis on certain distinctive subsets of F&V varieties that show superior outcomes (10, 11). For maximizing dietary antioxidant nutrient selection, the evaluations of F&V pinning on antioxidant profile and functional activity are applicable for mortality risk reduction, in particular that of CVD and cerebrovascular disease (12).

Fruits grown in tropical areas such as Thailand and Southeast Asia have long been used as functional foods and natural remedies (13). For example, papaya and pomelo enhance the digestive system function and the latter also serves as an antiflatulent. The uses of longan fruit have been extended from tonic and memory boosting to the prebiotic activity of the pulp-derived polysaccharides (14, 15). Tropical fruits and other fruits and vegetables are rich in vitamins and phytochemicals such as phenolics and flavonoids, which show various health benefits, including antihypertensive effects and endothelial function improvement (16, 17). Therefore, in this study, we assessed the antioxidant capacities of a selection of 10 regional fruits, analyzed their phytochemical contents, and tested for their inter-assay correlations. To study these fruit samples that could potentially protect endothelial cells from oxidative stress, endothelial cells were exposed to H_2_O_2_ and evaluated for cell survival. Moreover, we investigated the effect of these fruits on endothelial NO production and wound healing.

## Materials and methods

### Materials

All chemicals were purchased from Sigma-Aldrich (https://www.sigmaaldrich.com/) or otherwise indicated. The human endothelial cell line, EA.hy926 was obtained from ATCC^®^ (cat.# CRL-2922™; https://www.atcc.org/). All the cell culture supplies were purchased from Gibco^®^ and Life Technology^®^ (ThermoFisher Scientific, https://www.thermofisher.com/), including the Dulbecco’s Modified Eagle Medium (DMEM) culture media, fetal bovine serum (FBS), trypsin 2.5%, and penicillin-streptomycin (10,000 U/mL). Cell culture flasks and pipette tips were purchased from Corning^®^ Costar^®^ (A.N.H. Scientific Marketing, Bangkok, Thailand). All standard chemicals for high performance liquid chromatography (HPLC) analysis, including ellagic, gallic, and chlorogenic acid, were purchased from Sigma-Aldrich, USA.

### Fruit extract preparation

The 10 tropical fruits used in this study, comprising *Ananas comosus* L. (pineapple, AC), *Annona squamosa* L. (sugar apple, AS), *Carica papaya* L. (papaya; unripe, CP-u; ripe, CP-r), *Dimocarpus longan* Lour. (longan, DL), *Garcinia mangostana* L. (mangosteen, GM), *Litchi chinensis* Sonn. (lychee, LC), *Lansium domesticum* Corrêa. (langsat, LD), *Mangifera indica* L. (mango; unripe, MI-u; ripe, MI-r), *Nephelium lappaceum* L. (rambutan, NL) and *Psidium guajava* L. (guava, PG), were purchased from a local fruit market in Thailand. As papaya and mango are consumed both unripe and ripe, both stages of these fruits were investigated. The selected fruits were extracted under semi-sterile conditions. Briefly, fruits were washed and peeled, only the fresh pulp was collected, and cut into small pieces. Then, the fruit juices were obtained using a compact juice extractor (Braun MP75 Multipress Compact). The juices were filtered through a sterile Whatman No.1 filter paper applying sterile filtration equipment. The filtrates were then dried by freeze-drying. The dry powders were stored at −40°C until further use. For the assessments of phenolic and flavonoid contents and all other antioxidant capacity assays, the fruit powders were dissolved in the same buffers as the standard references in each assay.

### Phenolic and flavonoid content evaluation

#### Total phenolic content (TPC)

The phenolic content was evaluated by using the Folin–Ciocalteu assay (18). Briefly, 100 μL of the Folin–Ciocalteu reagent (Sigma-Aldrich) was added to each well of a 96-well plate. Next, 20 μL of each sample or standard gallic acid were added in the wells and mixed. Then, 300 μL of 7.5% sodium carbonate solution was added into the wells and the plate was incubated at 40 °C for 30 min. The solution was then cooled down at room temperature for 5 min and the absorbance of each well was measured at 756 nm using a spectrophotometer (SpectraMax^®^ M2e microplate reader, Molecular Devices, USA). The phenolic content in each sample was presented as the gallic acid equivalent (μg gallic acid/mg dry weight).

#### Total flavonoid content (TFC)

The flavonoid content was evaluated using an aluminum colorimetric assay with minor modifications (19). Briefly, 100 μL of each sample or standard catechin were added to a 96-well plate. Then, 6 μL of 5% NaNO_2_ in distilled water was added to each well and the plate was incubated for 5 min. Next, 6 μL of 10% AlCl_3_ in distilled water was added to each well and the plate was incubated for 1 min followed by the addition of 40 μL of NaOH with volume adjustment to 200 μL. The readings of the samples were taken at the absorbance of 510 nm and the flavonoid contents were calculated based on the extrapolation from the catechin standard curve. Data are presented as μg catechin/mg dry weight (DW).

### Antioxidant capacity evaluation

#### Ferric reducing antioxidant power (FRAP) assay

The FARP assay was performed based on the reduction of Fe^3+^ to Fe^2+^ as described previously (20). Briefly, The FRAP reagent mixture (1 mM TPTZ, 2 mM FeCl_3_ in 40 mM HCl, and 30 mM acetate buffer, pH 3.6), was mixed with various concentrations of samples and the FeSO_4_ standard, then incubated at room temperature for 5 min. The product of the Fe^3+^ complex oxidation generates the Fe^2+^ complex, which was measured at 593 nm. Extrapolated from the FeSO_4_ standard curve, the FRAP values were calculated and presented as μmol FeSO_4_ per mg dry weight (μmol FeSO_4_/mg DW).

#### Oxygen radical absorbance capacity (ORAC)

ORAC was noted as described previously (21). Briefly, 100 μL of 10 nM 2′,7′-dichlorofluorescin diacetate (DCF, Sigma-Aldrich) was dissolved in 75 mM KH_2_PO_4_ and added into each well of 96-well plates. Next, 25 μL of various concentrations of samples and standard Trolox were added into the wells and the plates were incubated at 37 °C for 5 min. Then, 25 μL of 165 mM AAPH was added into the wells. The fluorescent intensities of the samples in each well were measured for 60 min at 1-min intervals at the excitation/emission wavelengths of 485/528 nm. The area under the curve (AUC) was calculated and the net AUC (AUC of the sample − AUC of blank) was used to evaluate the antioxidant capacity compared with the reference Trolox standard (μmol Trolox/mg DW).

### Specific ROS-scavenging activity evaluation

#### Hydrogen peroxide (H_2_O_2_) scavenging activity assay

The assay for scavenging activities against H_2_O_2_ was performed as described previously (21), with minor modifications. Briefly, the mixture, containing 125 μM of homovanillic acid (HVA) and 0.1 U of horseradish peroxidase in 50 mM KH_2_PO_4_ pH 7.4, was mixed with various concentrations of samples or standards. To start the reaction, 30 μM H_2_O_2_ was added to the mixtures and incubated at room temperature for 30 min. When the HVA dimer was formed it was measured at the excitation/emission wavelengths of 315/425 nm using a spectrophotometer (SpectraMax^®^ M2e microplate reader, Molecular Devices, USA). The concentrations of the fruit extracts that resulted in 50% inhibition of HVA formation were calculated and presented as IC_50_ values in μg/mL.

#### Hydroxyl radical (OH) scavenging activity

This assay was performed as described previously (21), with minor modifications. Briefly, the assay buffer, containing 0.2 mM L-ascorbic acid, 2 mM EDTA, 0.2 mM H_2_O_2_, 1.12 mM 2-deoxy-2-ribose and 0.1 mM FeCl_3_, was mixed with various concentrations of each fruit extract or the Trolox standard and incubated at 50 °C for 20 min. The trichloroacetic acid solution was added to the mixture to achieve a final concentration of 1.12%, followed by the addition of a 2-thiobarbituric acid solution to achieve the final concentration of 0.4%. The mixtures were incubated at 95 °C for 15 min. The pink color products representing thiobarbituric acid reactive substances (TBAR) were measured at the absorbance (A) of 550 nm. The inhibition percentage of each sample was calculated using the following equation:

$$\%\text{inhibition }{=\;(\text{A}}_{\text{blank}}\;-\;{\text{A}}_{\text{sample}})\;\times \;100/({\text{A}}_{\text{blank}}),$$ 1)

#### Hypochlorous acid (HOCl) scavenging activity

The assay was performed as described previously (21). First, the solutions of 40 μM TNB and 40 μM NaOCl were prepared using the molar absorption coefficients of 13,600 and 100 M^−1^cm^−1^, respectively. Next, various concentrations of samples or the ascorbic acid standard were mixed with TNB in a 1:1 ratio and the absorbance (A) was measured at 412 nm. Then, 40 μM of NaOCl was added to the mixture in a 1:2 ratio and incubated for 5 min. The percentage of residual TNB was calculated as % TNB inhibition using the following equation:

$$\%\text{ TNB remaining }=\text{ 100 }-\;{(\text{A}}_{\text{before}}\;-\;{\text{A}}_{\text{after}})\;\times \;100/{\text{A}}_{\text{before}},$$ 2)

#### Superoxide (O_2_^−^) scavenging activity assay

As described previously (21), the assay began with the preparation of a reaction mixture containing a sample or the reference ascorbic acid standard mixed with 77.4 μM nitrotetrazolium blue (NBT), and 90 μM NADH in 19 mM KH_2_PO_4_. Phenazine methosulfate (PMS) was added to the mixture to achieve the final concentration of 9 μM, followed by further incubated at room temperature for 3 min. The reaction of PMS and NADH generates O2•¯ that coverts NBT to NBT formazan, which can be monitored at 560 nm. The scavenging activity is presented as the inhibition percentage of O_2_^−^ generation.

### HPLC analysis of common phytochemicals

Four phytochemicals (chlorogenic acid, ellagic acid, gallic acid, and quercetin) were quantified using an HPLC analysis system (Shimadzu chromatographic system, Shimadzu, Japan). The HPLC system consists of an LC pump (LC-20AD), autosampler (SIL-20AC HT), HPLC column oven (CTO-20A), a photodiode array detector (SPD-M20A), system controller (CBM-20A) and a Shimazu LCsolution^®^ software. An Inertsil^®^ ODS-3 analytical column 5020-01732 (reversed-phase C18 column) was used for the analysis (22). The HPLC-grade chemical standards and fruit extract samples were prepared in 1 mL of Milli-Q^®^ type 1 ultrapure water (Merck Millipore, USA) followed by sonication for 30 sec, filtered through a 0.45 μm nylon filter, and kept on ice throughout the process to protect the biomolecules from degradation. The mobile phases and detection wavelengths are shown in Table 1. Several solvent combinations were developed and optimized to obtain good separation of peaks and symmetry of peak shapes referring to Sawant et. al. (23). The determination of each of the phytochemical contents was extrapolated from HPLC standard curves ranging between 0 and 100 μg/mL. Each sample was injected at least three times and the means of data points were presented.

**Table 1:** HPLC conditions for the analysis of phytochemicals.

Standard	Mobile phase	Detection λ (nm)	Retention Time (min)
Ellagic acid	20 mM phosphate buffer pH 2.5 and acetonitrile (70:30); isocratic elution, flow rate 1.0 mL/min	254	4.945
Gallic acid	20 mM phosphate buffer pH 2.5 and acetonitrile (95:5); isocratic elution, flow rate 1.5 mL/min	270	4.805
Quercetin and Chlorogenic acid	20 mM phosphate buffer pH 2.5 (component A) and acetonitrile (component B); gradient elution, flow rate 1.5 mL/min 0–3 min: 10% B 4–15 min: 10–90% B 16–21 min: 90% B 21–22 min: 90–10% B 22–30 min: 10% B.	255 and 325, respectively	12.677 and 8.626, respectively

### Cell culture

The human endothelial cells EA.hy926 were cultured in DMEM supplemented with 10% FBS and 100 U/mL of penicillin–streptomycin antibiotics. Cells were incubated at 37 °C in a humidified 5% CO_2_ atmosphere. The cell culture media were changed every 3 days throughout the experiments. Cells were subcultured to new culture flasks when their confluence reached approximately 80%. To perform each experiment, EA.hy926 at a density of 1 × 10^4^ cells/well was cultured in a 96-well plate for 18–24 h and preincubated with different concentrations of fruit extracts (10, 100, and 1,000 μg/mL) diluted in cell culture media for 48 h.

### Cell viability assay

The cell viability was evaluated using the MTT assay as described previously (21). Following the incubation with the fruit extracts for 48 h, the cell culture media were removed and replaced with fresh media containing 300 μM H_2_O_2_, then further incubated for 2 h. Then, fresh media containing 0.25 mg/mL MTT was added to each well and incubated for 3 h. The formazan crystals were generated and dissolved in DMSO for the absorbance readings at 550 nm, which corresponded to the degree of cell viability. The fruit samples that induced a significant increase in cell viability were selected for the upcoming endothelial wound healing assays.

### Determination of NO production

The effect of the fruit extracts on the NO production in the endothelial cells was determined using the 2,3-diaminonaphthalene (DAN) assay as described previously (24). Briefly, endothelial cells were pretreated with various concentrations of fruit extracts and incubated for 48 h. The supernatant of each sample was centrifuged at 2,000 × *g* for 1 min and transferred to 96-well plates. Next, 10 μL of 50 μg/mL DAN, dissolved in 0.62 N HCl, was added into each well and incubated at room temperature for 10 min. The reaction was stopped by the addition of 5 μL of 2.8 N NaOH. The EA.hy926 cell-derived NO release was measured by detecting the fluorescence intensity at the excitation/emission of 360/440 nm compared with that of NaNO_2_ (reference standard). The fruit samples that induced a significant increase in NO production were selected for the upcoming endothelial wound healing assay.

### Endothelial wound healing assay

The effect of fruit extracts on wound healing was evaluated through the scratch-wound assay (25). Briefly, EA.hy926 cells were seeded at a density of 1 × 10^6^ cells/well in a 6-well cell culture plate and cultured for 18–24 h until reaching confluence. The cell layer was then scratched using a 200-μL pipette tip. The media were removed and treated with fruit extracts for 12 and 24 h. After several rounds of PBS wash, images were taken using an inverted light microscope (Olympus, Japan). The percentage of wound closure was calculated using the Cell^B software (Olympus, Japan). The fruit samples that induced a significant increase in wound closure were selected for the upcoming Transwell cell migration assay.

### Transwell cell migration assay

The cell migration assay was conducted as described previously (26). Briefly, EA.hy926 cells at a density of 2 × 10^4^ cells/mL were seeded in 8-μm pore sized cell culture inserts and cultured in DMEM containing 0.5% FBS in a 24-well plate. Fruit extracts or VEGF (25 ng/mL, positive control) were added into the plates, which were then incubated for 12 h. Following the incubation, the medium was removed from the cell inserts, and cells were fixed with 3.7% formaldehyde at room temperature for 2 min, followed by several round of PBS wash. Cells were permeabilized with 100% methanol at room temperature for 20 min. The migrated cells were stained with 0.1% crystal violet for 15 min. Then, the dye was removed and washed twice with PBS. The attached cells on the upper chamber were removed by a cotton swab. The migrated cells were observed under an inverted microscope (Olympus, Japan). The images were captured with a digital camera (Olympus, Japan). The relative numbers of cell migration in each treatment group were compared with the vehicle-treated control group.

### Tube formation assay

This assay was conducted following the previously-reported procedures (27). The Matrigel matrix (Corning^®^, https://www.corning.com/) was thawed overnight at 4 °C. Then, a pre-chilled 24-well plate was coated with Matrigel and incubated at 37 °C for 30 min. The EA.hy926 suspensions at the density of 9 × 10^4^ cells in DMEM, containing 0.5% FBS and 25 ng/mL fruit extract or VEGF (25 ng/mL), were seeded onto each well of a 24-well plate coated with Matrigel and incubated at 37 °C for 12 h. After the incubation, three randomized files were visualized and images were captured under an inverted microscope (Olympus, Japan). The tube length formation was measured and calculated using the Cell^B software (Olympus, Japan).

### Statistical analysis

Data are presented as the mean ± SEM and were analyzed using the Graphpad Prism Ver. 5.0 software. The statistical significance was determined at *P* < 0.05 using one-way analysis of variance (ANOVA) with Turkey’s test for post hoc analysis. The Pearson’s correlation coefficient was applied to test the relationship between two values of antioxidant contents and/or ROS-scavenging assays.

## Results

### TPC and TFC content in the fruit extracts

A broad range of TPC and TFC contents in fruit extracts is shown in Table 2. Among all fruit extracts, GM contained the lowest amount of TPC at 1.18 + 0.04 mg gallic acid/g DW whereas the highest TPC content was found in LD at 7.90 + 0.43 mg gallic acid/g DW. The lowest and highest amounts of TFC were detected in LC and PG at 0.06 + 0.06 and 1.56 + 0.09 mg catechin/g DW, respectively.

**Table 2:** Fruit extract total flavonoid content (TFC), total phenolic content (TPC), and reactive oxygen species (ROS) scavenging activities

Sample	TPC (mg gallic acid/g dry weight [DW])	TFC (mg catechin/g DW)	Total antioxidant capacity		ROS-scavenging activity			
			Ferric reducing antioxidant power (FRAP) (μmol Trolox/g DW)	Oxygen radical absorbance capacity (ORAC) (μmol Trolox/g DW)	Hydroxyl radical (IC_50_, μg/mL)	Hypochlorous acid (IC_50_, μg/mL)	H_2_O_2_ (IC_50_, μg/mL)	O_2_^•–^ (IC_50,_ μg/mL)
Trolox					45.89 ± 3.21	-	0.58 ± 0.05	-
ASC					-	93.37 ± 5.34	-	357.05 ± 2.19
AC	2.09 ± 0.08^ab^	0.21 ± 0.02^a^	14.40 ± 0.62^bc^	33.55 ± 2.09^b^	457.13 ± 15.15^abc^	5851.61 ± 25.97^b^	217.10 ± 30.57^bcd^	559.21 ± 35.39^bc^
AS	7.19 ± 0.91^de^	1.22 ± 0.11^d^	18.04 ± 0.62^c^	30.88 ± 2.21^ab^	266.24 ± 18.75^d^	1503.02 ± 40.00^g^	89.09 ± 64.85^cd^	1396.39 ± 231.76^abc^
CP-u	3.54 ± 0.06^abc^	0.20 ± 0.02^ab^	14.62 ± 0.87^bc^	48.10 ± 1.23^c^	280.17 ± 3.57^d^	1316.63 ± 21.78^g^	196.39 ± 7.36^bcd^	846.53 ± 40.16^bc^
CP-r	5.10 ± 0.72^cd^	0.57 ± 0.08^c^	58.07 ± 1.69^e^	51.01 ± 2.93^c^	435.46 ± 25.55^ab^	2139.66 ± 47.66^ef^	93.99 ± 9.34c^d^	1365.72 ± 205.32^abc^
DL	3.99 ± 0.08^bc^	0.38 ± 0.03^bc^	34.01 ± 0.81^d^	38.62 ± 0.67^b^	371.66 ± 4.61^cd^	1399.62 ± 29.76^fg^	52.59 ± 23.14^d^	1268.12 ± 165.56^abc^
GM	1.18 ± 0.04^a^	0.32 ± 0.05^b^	11.79 ± 0.40^abc^	21.44 ± 1.67^a^	421.20 ± 3.64^abc^	7161.58 ± 415.22^b^	437.24 ± 34.68^b^	1770.41 ± 546.05^abc^
LC	1.45 ± 0.02^a^	0.06 ± 0.06^a^	8.06 ± 1.30^a^	23.13 ± 0.98^a^	389.61 ± 24.66^bc^	8696.30 ± 779.84^a^	805.39 ± 22.16^a^	2306.92 ± 618.18^a^
LD	7.90 ± 0.43^e^	0.37 ± 0.01^bc^	10.15 ± 0.00^ab^	16.27 ± 2.69^a^	434.64 ± 11.66^abc^	5983.49 ± 26.86^b^	346.85 ± 19.30^bcd^	1845.25 ± 263.01^ab^
MI-u	7.03 ± 1.89^cde^	0.28 ± 0.01^ab^	17.43 ± 1.40^c^	22.57 ± 0.74^a^	432.58 ± 6.59^abc^	3407.47 ± 50.20^d^	505.16 ± 156.72^ab^	1380.90 ± 113.63^abc^
MI-r	2.02 ± 0.09^ab^	0.42 ± 0.01^bc^	13.40 ± 0.81^bc^	21.83 ± 1.16^a^	499.04 ± 10.52^a^	3073.66 ± 70.45^de^	261.76 ± 9.39^bcd^	1426.47 ± 153.62^abc^
NL	4.98 ± 0.70^cd^	0.17 ± 0.01^ab^	14.20 ± 0.41^bc^	15.58 ± 1.40^a^	417.18 ± 18.10^abc^	4034.92 ± 156.06^cd^	406.07 ± 40.79^bc^	1227.32 ± 181.57^abc^
PG	3.45 ± 0.05^abc^	1.56 ± 0.09^e^	68.77 ± 0.41^f^	69.62 ± 1.94^d^	470.53 ± 53.39^ab^	2069.24 ± 24.76^ef^	119.84 ± 24.45^cd^	709.55 ± 41.11^bc^

**Note:** Data are shown as the mean ± SEM. The ANOVA Tukey post hoc test was used to determine the statistical significance at *P* < 0.05. Superscripts indicate significant differences in the means of samples in the same column while the same letters in the same column indicate no significant difference. DW: dry weight.

### ROS-scavenging activity

Table 2 shows total antioxidant capacities (FRAP and ORAC) and the specific ROS-scavenging activities. The highest total antioxidant capacity was found in PG for both FRAP and ORAC detection methods. The top-ranking of ROS-scavenging activities (the lowest IC_50_s) for OH^•^, HOCl, H_2_O_2_, and O_2_^−^ were demonstrated by AS, CP-u, DL, and AC, respectively. Interestingly, the scavenging activity of AC against O_2_^−^ is 1.6-fold of that of the reference antioxidant ascorbic acid (ASC). MI-r showed the lowest OH^•^ scavenging activity among the fruit samples while LC showed poor activities for the rest of ROS-scavenging tests, that is, HOCl, H_2_O_2_, and O_2_^−^.

**Fig. 1:**
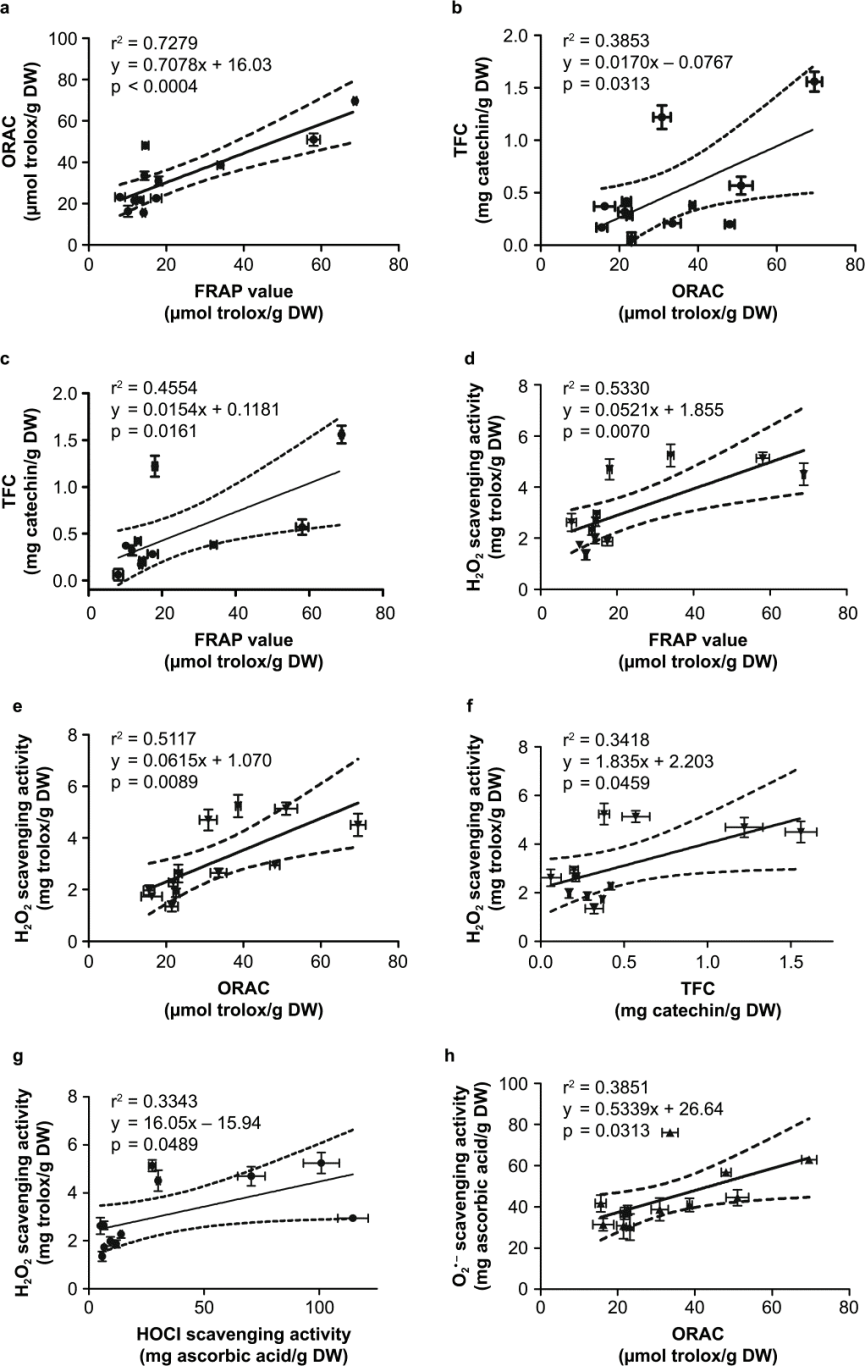
The correlations TFC, TPC, and ROS-scavenging activities. (**a**) FRAP vs. ORAC; (**b**) FRAP vs. H_2_O_2_; (**c**) ORAC vs. H_2_O_2_; (**d**) HOCl vs. H_2_O_2_; (**e**) ORAC vs. O_2_^−^. Pearson’s correlation analysis was used to evaluate significant correlations at *P* < 0.05.

There were eight significant correlations among the antioxidant activities which arre inter-related (Fig. 1). The two common antioxidant capacity assays, FRAP and ORAC, showed a strong positive association at *r*^2^ = 0.7279, *P* < 0.001 (Fig. 1a), and these two assays corresponded well to TFC (Fig. 1b, 1c). H_2_O_2_ scavenging activity prominently correlated well with other antioxidant evaluations, including FRAP, ORAC, TFC, and HOCl scavenging activities (Fig. 1d–g) while superoxide scavenging activity had a positive correlation only to ORAC (Fig. 1h). Interestingly, TPC and hydroxyl radical scavenging activity did not show any correlation to other antioxidant capacities tested in this study.

**Fig. 2:**
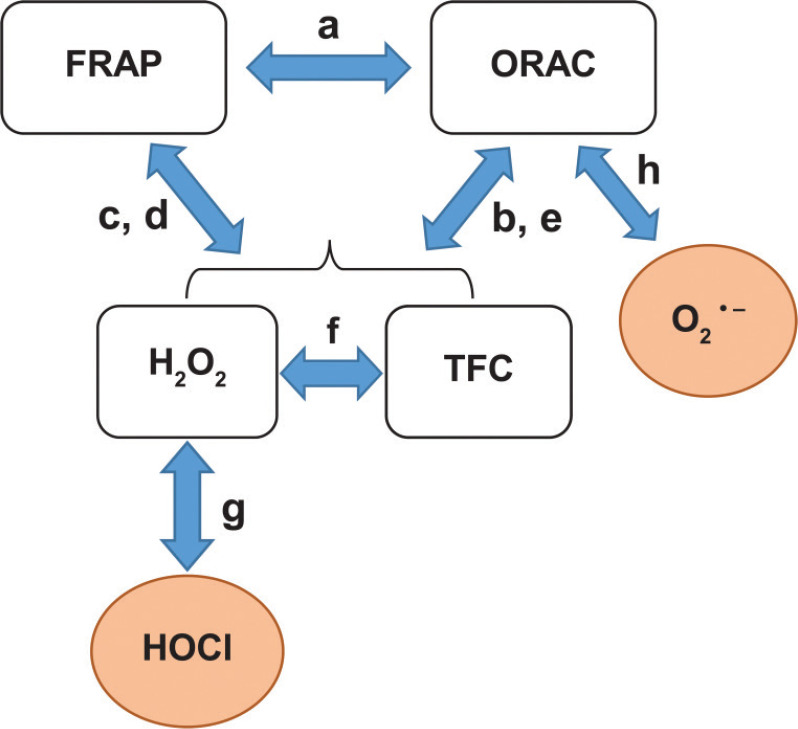
The diagram demonstrating the relationships among eight antioxidant evaluations. Abbreviations: FRAP, ferric reducing antioxidant power; ORAC, oxygen radical absorbance capacity; TFC, total flavonoid content. The letters indicated along the arrows refer to significant relationships according to the subset of graphs in Fig. 1.

The relationships of TFC, TPC, and ROS-scavenging activities can be depicted as a diagram shown in Fig. 2. The letters along the arrows indicate the association graphs demonstrated in Fig. 2.

### HPLC analysis of the phytochemical contents

**Fig. 3:**
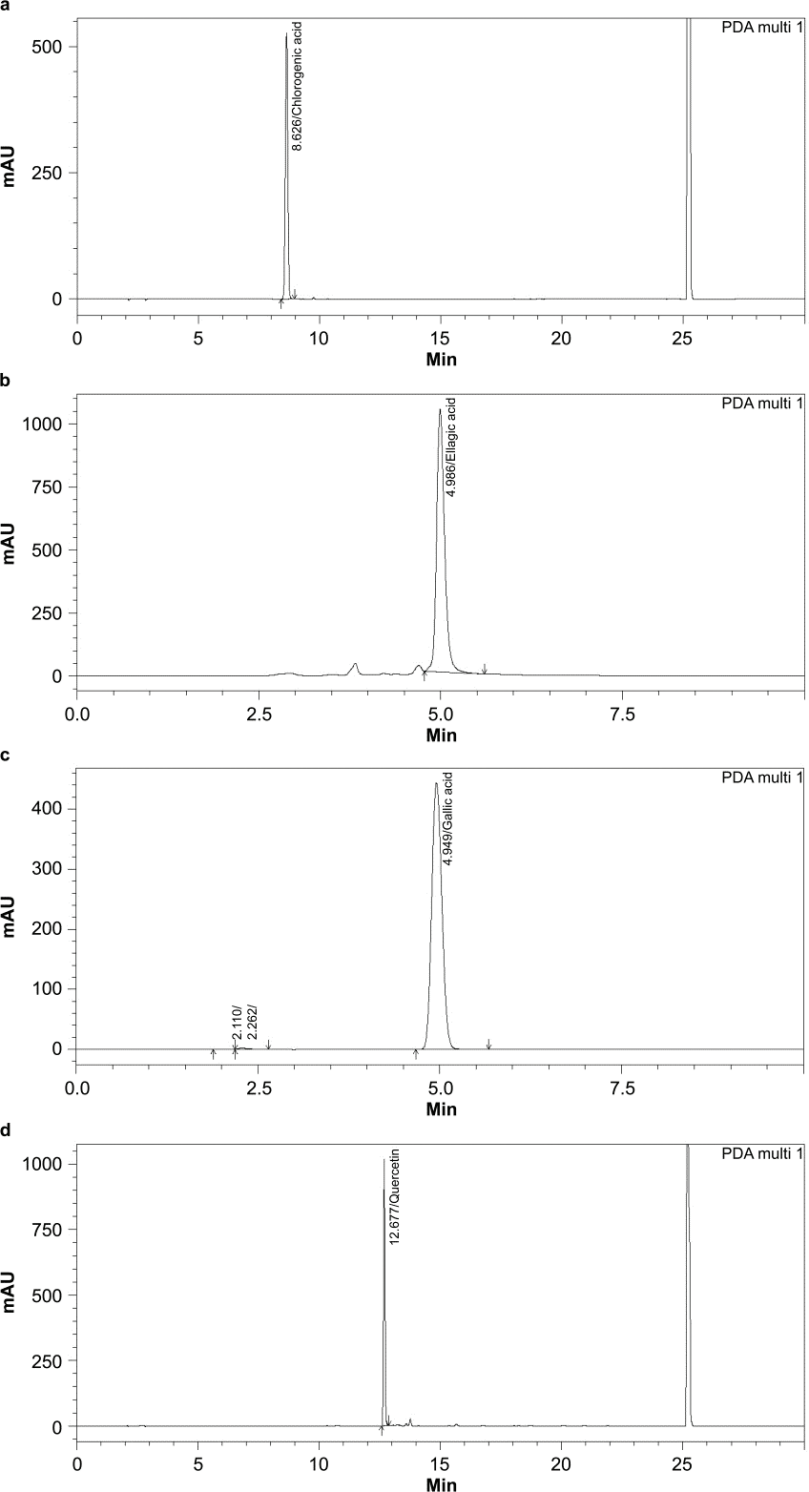
HPLC chromatogram and standard curves of phytochemical contents in fruit extracts. (**a**) chlorogenic acid, (**b**) ellagic acid, (**c**) gallic acid, and (**d**) quercetin.

The HPLC chromatograms and standard curves of four phytochemicals, including chlorogenic acid, ellagic acid, gallic acid, and quercetin are shown in Fig. 3. The contents of phytochemicals per 1-g dry powder of fruit extracts are shown in Table 3. The two common phytochemicals gallic acid and quercetin were detected in all fruit samples and the highest amount was found in AC and MI-u, respectively. Notably, the bioactive phytochemicals like ellagic acid could not be detected in all fruit samples while chlorogenic acid was found only in AC, CP-r, and PG.

**Table 3:** HPLC quantification of four phytochemical contents, common in all fruit samples

Fruit	Phytochemical content (μg/g dry weight)		
	Chlorogenic acid	Gallic acid	Quercetin
AC	7.91 ± 0.07	74.99 ± 11.05	1.75 ± 0.04
AS	N/D	14.86 ± 13.40	4.41 ± 1.96
CP-u	N/D	2.03 ± 0.38	1.87 ± 0.04
CP-r	5.38 ± 0.03	5.72 ± 1.41	1.98 ± 0.02
DL	N/D	2.28 ± 0.11	2.26 ± 0.01
GM	N/D	0.97 ± 0.03	2.17 ± 0.03
LC	N/D	33.84 ± 14.11	10.83 ± 0.01
LD	N/D	6.72 ± 1.31	2.19 ± 0.01
MI-u	N/D	58.55 ± 1.08	19.85 ± 0.02
MI-r	N/D	39.31 ± 1.55	16.75 ± 0.01
NL	N/D	11.29 ± 2.08	5.84 ± 0.02
PG	4.03 ± 0.01	29.17 ± 6.61	14.12 ± 0.05

Note: N/D, not detectable.

**Fig. 4:**
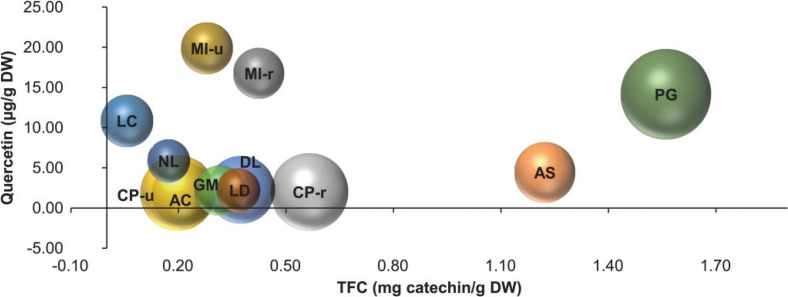
Three-dimension plots of antioxidant activities of fruit extracts. The plot represents the relationship of total flavonoid content (TFC, x-axis), quercetin (y-axis), and ORAC activity (bubble size) of fruit extracts per gram dry weight (DW).

Based on the positive relationship between ORAC and TFC, we further explored whether the amounts of quercetin, a common flavonoid found in all fruit extracts, could have any association with TFC and ORAC. Figure 4 shows a three-dimensional bubble plot of these three parameters. Although quercetin shows no significant relationships between ORAC or TFC (*r*^2^ = 0.0021 and 0.0286, respectively); the plot depicts that PG is outstanding for all three parameters seconded by AS. Among all fruit samples, mango in both stages of ripening contains the highest amount of quercetin.

### Fruit extract-mediated prevention of H_2_O_2_-induced endothelial cell death

**Fig. 5:**
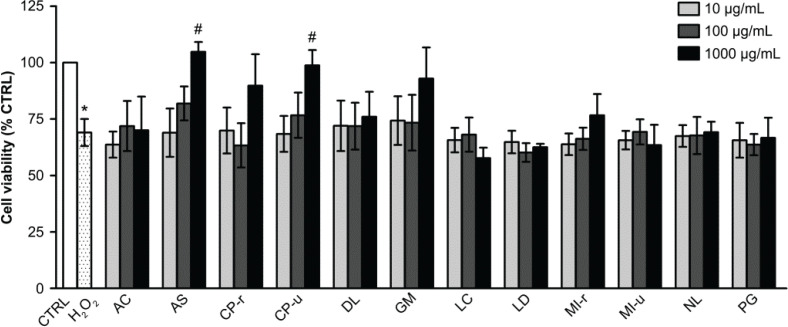
The preventive effect of fruit extracts on H_2_O_2_-induced EA.hy926 cell death. EA.hy926 cells were pretreated with various fruit extracts and then incubated with 300 μM H_2_O_2_ for 2 h as described in the Materials and Methods section. Data are shown as mean ± SEM, * *P* < 0.05 when compared with the vehicle control group (C), # *P* < 0.05 when compared with 300 μM H_2_O_2_ treated group.

H_2_O_2_ significantly decreased cell viability to 66.50 ± 3.79% when compared with the control group (*P* < 0.01). Of all extracts, only AS and CP-u pretreatment at 1,000 μg/mL showed a cytoprotective effect against H_2_O_2_ insult. After 24 h of H_2_O_2_ exposure, the viability of cells incubated with AS and CP-u significantly recovered to 104.79 ± 4.31% and 98.76 + 6.80%, respectively (Fig. 5). The cytoprotective or proliferative effects of AS and CP-u on endothelial cells were carried on to further experiments related to wound healing.

### The effect on NO release in EA.hy926 cells

**Fig. 6:**
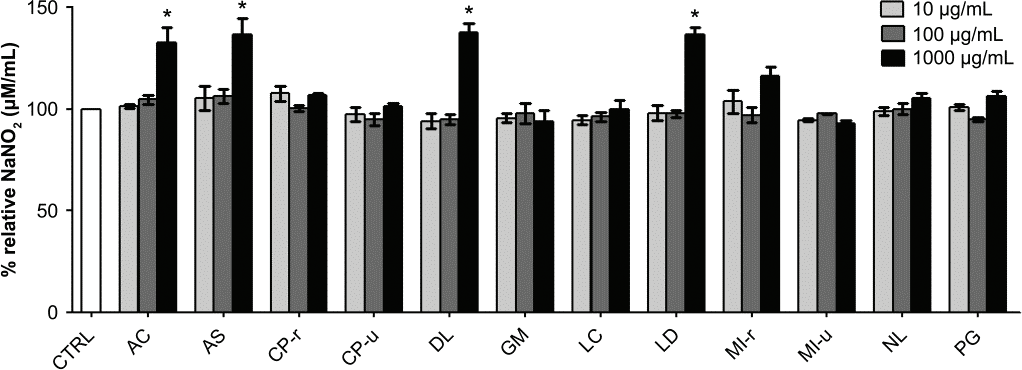
Effect of fruit extracts on NO production. Endothelial cells were incubated with various concentrations of fruit extracts (indicated in the graph) for 48 h. Data are presented as percent relative NaNO_2_ formation (mean ± SEM). * *P* < 0.05 when compared with the vehicle control group (CTRL).

Among the three concentrations of fruit extracts used for cell incubation (10, 100, and 1,000 μg/mL), only the concentration at 1,000 μg/mL of four extracts significantly enhanced NO production, representing the nitrite levels produced by the endothelial cells. AC, AS, DL, and LD elevated nitrite productions to 132.6 ± 6.1%, 136.6 ± 6.5%, 137.7 ± 3.5%, and 136.6 ± 3.0%, respectively (Fig. 6). As NO is closely related to wound healing and cell migration, these four fruit extracts were selected for further experiments.

### The effect of the fruit extracts on endothelial wound healing

**Fig. 7:**
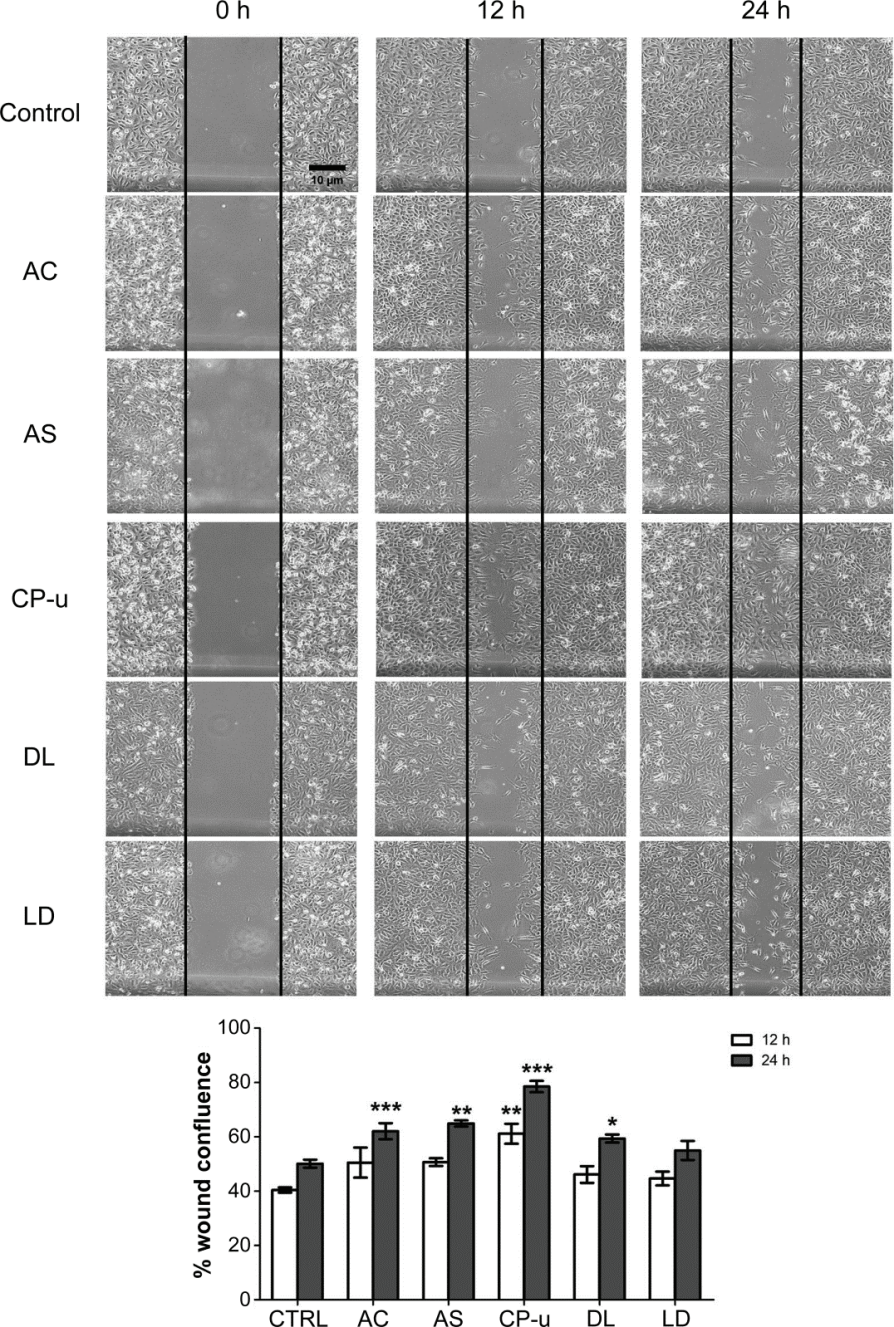
The endothelial wound healing effect of fruit extracts. Scratch wounds were initiated at 0 h and measured at 12 and 24 h. Photographs of wound closure were taken at each time point and calculated as % wound confluence. Data are presented as mean ± SEM. *, *P* < 0.05; **, *P* < 0.01; ***, *P* < 0.001 compared with vehicle-treated group (CTRL).

Five fruit extracts were selected based on their properties of inducing cell survival against H_2_O_2_-induced oxidative stress and enhancing NO production. Their effects on wound healing were primarily evaluated by cell scratch assay (Fig. 7). At 12 h after the cell scratch, none of the treatment groups showed any significant difference in wound confluence when compared with the control group (40.43 ± 0.97% wound closure). However, when evaluated at 24 h, four fruit extracts (AC, AS, CP-u, and DL at 500 μg/mL) significantly increased wound closure to 62.08 ± 2.97%, 64.93 ± 1.19%, 78.486 ± 2.069%, and 59.37 ± 1.49%, respectively. LD did not improve endothelial wound closure. Therefore, only four fruits were selected for further experiments.

### The effect of the selected fruits on endothelial cell migration

**Fig. 8:**
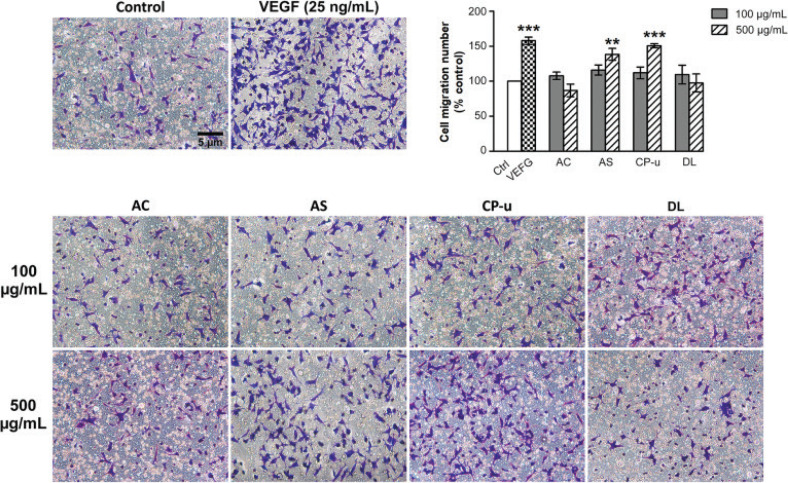
Effect of fruit extracts on cell migration. EA.hy926 cells were seeded onto the insert chambers and the migrated cells were stained with 0.1 % crystal violet. Photographs were collected and the migrated cells were counted as described in the Materials and Methods section. Data are presented as mean ± SEM. ** *P* < 0.01, and *** *P* < 0.001 compared with the vehicle control (Control, Ctrl).

Endothelial cell mobility through Boyden chambers was stimulated by VEGF (25 ng/mL) or fruit extracts. VEGF enhanced cell migration to 157.88 ± 5.13% (*P* < 0.001) when compared with the vehicle control group (Fig. 8). AS and CP-u at 500 μg/mL significantly enhanced cell migration to 138.46 ± 8.46% and 150.83 ±2.97%, respectively, while AC and DL did not alter cell mobility.

### The effect of the selected fruit extracts on capillary-like tube formation

**Fig. 9:**
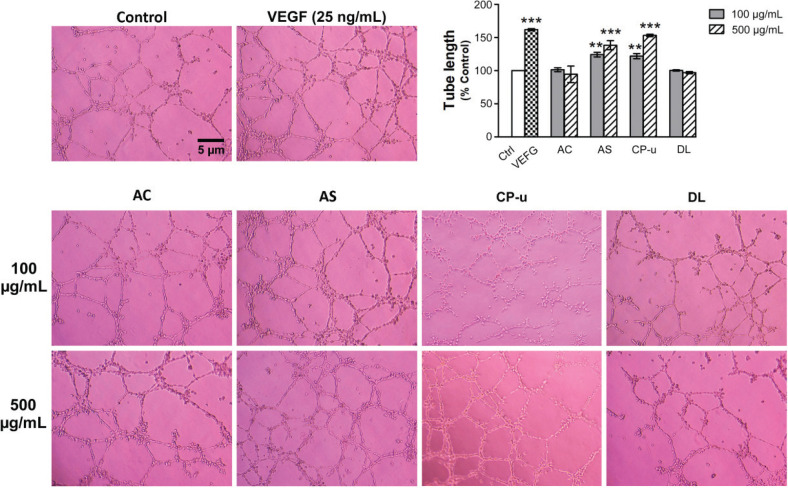
The *in vitro* angiogenic effect of fruit extracts. Matrigel was used as a 3D matrix to produce capillary-like tube formation. After 12 h, tube lengths were observed, and photographs were taken under an inverted microscope as described in the Materials and Methods section. Data are presented as mean ± SEM. ** *P* < 0.01 and *** *P* < 0.001 when compared with vehicle-treated control (Control, Ctrl).

As shown in Fig. 9, the positive control VEGF at 25 ng/mL significantly increased tube formation to 161.87 ± 1.59% (*P* < 0.001) when compared with the vehicle control group. AS at 100 and 500 μg/mL induced a significant increase of tube formation by 124.19 ± 3.52% and 138.39 ± 6.84%, respectively. The lengthening of the capillary-like tube was markedly observed in cells treated with CP-u at 500 μg/mL, which enhanced the tube formation to 153.20 ± 1.76. In contrast, AC and DL did not induce any significant change in tube formation.

## Discussion

The role and functional effect of bioactive phytochemicals in cardio- and cerebrovascular disease prevention are undebatable (11, 12). Tropical fruits contain high amounts of antioxidants including phenolics, flavonoids, terpenes, and other compounds, accounting for the reduction of cellular oxidative stress that contribute to CVD pathogenesis (28, 29). The 10 regional fruits cultivated in Thailand differentially showed a wide range of antioxidant capacity and specific ROS-scavenging activity. The functional screening assays for oxidative stress cytoprotection, along with the NO release stimulation, resulted in five fruits (AC, AS, CP-u, DL, and LD) that were further investigated in the endothelial wound healing assays. Only three fruit extracts, including AC, AS, and CP-u, improved endothelial wound healing. The two fruit extracts, AS and CP-u, correspondingly affirmed endothelial cell migration and capillary-like tube formation. Therefore, AS and CP-u are selective functional fruits that protected against oxidative cell death and enhanced endothelial wound healing.

ROS-induced endothelial dysfunction is driven by the destruction of NO bioavailability and oxidative damage to macromolecules as well as triggering signal transduction pathways toward atherosclerosis and other CVDs (1). As a result, therapeutic implication or disease prevention approaches rely on using antioxidants with high ability to scavenge ROS, especially the species associated with the progress of endothelial dysfunction (i.e. O_2_·^–^, H_2_O_2_, OH, and HOCl (30). While TPC and TFC qualitatively represent antioxidant activity in samples obtained from natural resources, the ORAC assay evaluates the antioxidant capacity, often used as a reference value for nutraceutical products. However, many scientific reports apply various methods for their investigations, such as FRAP, DPPH, and TEAC assays (31). Among these assays, FRAP and ORAC could be commonly found in the literature used for the comparison of ROS capacities in samples derived from different sources of materials and preparations. The fruit extracts exhibited a good correlation between these two assays, which is in good accordance with other reports (32, 33). The new finding in this study is that the relationship between FRAP and ORAC is extended to the H_2_O_2_ scavenging activity and TFC but not to TPC. Further attempts to quantify bioactive phytoantioxidants reveal that all fruits contained a common phenolic and flavonoid, that is, gallic acid and quercetin, respectively. These two dietary natural phytochemicals are well-studied both *in vitro* and *in vivo* indicating a wide range of bioactivities, including anti-inflammation, antimicrobial, and antitumor activities (34, 35). A recent meta-analysis of randomized controlled trials for the effect of supplementation with quercetin on levels of plasma C-reactive protein reveals a significant reducing effect (36) as well as causing a significant reduction in blood pressure (37). However, it did not affect the plasma lipid profile (38). This suggests that quercetin exhibits more favorable impact on the anti-inflammatory activity and endothelial function compared to the potential metabolic complications. Recent review articles by Patel et al. (39) and Deng et al. (40) emphasize the protective role of quercetin in endothelial dysfunction from oxidative stress and its potential use as an antiatherosclerotic agent and in CVD prevention. Quercetin prevents endothelial dysfunction by competitively inhibiting myeloperoxidase-dependent HOCl generation and NADPH oxidase-dependent O_2_·^–^ production that play a crucial role in atherosclerosis (41, 42). Thus, our results are in line with the current evidence of using natural phytochemicals as a complementary remedy and in CVD prevention. Commonly found as an active ingredient in medicinal plants and fruit extracts, gallic acid (one of the bioactive compositions) shows numerous potential remedial effects such as in the case of diabetes, infection, and inflammation (43–46). The antihypertensive effect of gallic acid is evident through the reduction of NADPH oxidase, activation of NO synthesis by inhibiting eNOS degradation, and suppression of inflammation by reducing TNF-α, p38, ERK and NFkB phosphorylation (47–49). Therefore, the consumption of these tropical fruits is undoubtedly beneficial to cardiovascular health.

Chlorogenic acids are another extensively studied group as nutraceuticals, which we detected in three fruit extracts, including AC, CP-r, and PG. Chlorogenic acid is evident for biological activities of antioxidant, anti-inflammatory, glucose, and lipid metabolism regulation and its beneficial effect on CVD (50, 51). For instance, maoberry, an indigenous Thai fruit rich in chlorogenic acid, quercetin, and other phytoantioxidants, exhibited significant antioxidant stress effect in a high-fat diet rat model, leading to the inactivation of pro-inflammatory mediator release (e.g. tumor necrosis factor-alpha [TNF-α] and interleukin-6 [IL-6]), as well as the inhibition of adhesion molecule expression (e.g. vascular cell adhesion molecule-1 [VCAM-1] and monocyte chemoattractant protein-1 [MCP-1]) that participate in CVD progress and pathology (52, 53). A recent *in vivo* study shows enzyme inhibitory effects of AC against phosphodiesterase, monoamine oxidase, and angiotensin-converting enzyme activities in rat heart and brain homogenates (54). The mechanism of anti-inflammatory and immune modulation effects of CP-u is associated with the interactions with toll-like receptors subtypes 2, 4, 5, 3, and 9 (55). Therefore, these three fruits may exert CVD risk reduction, yet further studies are warranted.

Apart from the characteristics of the phytochemicals found in each type of fruit, the stages of fruit ripening also define the pattern of antioxidant activities. For example, Ataulfo mango, cultivated in Mexico, when ripe, contained 10.8, 1.4, and 2.3-fold-increased amounts of chlorogenic acid, vanillic acid, and protocatechuic acid compared to the fruit in the unripe stage, respectively (56). In contrast, during ripening, the mango cultivar ‘Khiao-sawoey’ grown in Thailand had a drop in gallic acid and quercetin contents by 0.7 and 0.8-fold, respectively. The TPC was reduced accordingly but TFC did not increase to 1.5-fold in the ripening stage. Unlike MI, CP-r exhibited increased amounts of both TPC (1.4-fold) and TFC (2.9-fold) as well as gallic acid (2.8-fold) and quercetin (1.1-fold) when compared to the unripe stage (CP-u). However, the cytoprotective effect against oxidative stress and the endothelial wound healing potential were observed only in CP-u. Therefore, considering a specific antioxidant content was not necessarily a determinant of health benefits without bioactivity tests.

As oxidative stress is a leading cause of endothelial dysfunction, which is a major risk factor and a predictor of CVD, improving endothelial function by minimizing oxidative stress is very important in reducing CVD risks. Among 10 fruit samples, AS and CP-u had both shown cytoprotective and endothelial wound healing effects independent of NO production. These fruit extracts had the greatest ability in removing OH (AS) and HOCl (CP-u), which significantly contributed to the reduction of cellular oxidative stress and the promotion of endothelial health. These findings are supported by a previous study, reporting that monotropein, a bioactive compound used in traditional Chinese medicine, promotes endothelial wound healing and angiogenesis by the inhibition of oxidative stress in endothelial progenitor cells (57). Moreover, individuals with normally low dietary F&V consumption had significant improvement of their endothelial function (measured by flow-mediated dilation) when provided with antioxidant-rich fruit juice for 6 weeks (16). Therefore, the assay for cytoprotection against oxidative stress is important for the screening of fruit samples that can promote endothelial health.

Even though single or purified phytochemicals have shown promising preclinical data, few agents are efficient in clinical studies. The antioxidant defense or redox regulation might be in balance due to different antioxidant compartments. The antioxidant activities of a single compound might accentuate only on particular antioxidant defense tiers. It is suggested that a combination of a wide variety of nutritional compositions is superior to influence CVD risk reduction rather than focusing on single bioactive nutrients (10). The failure to provide protective effects is often seen with large retrospective clinical studies when single antioxidant vitamins are consumed for such long periods of time (9). The balance of the antioxidant network can be disturbed and shifted toward ‘antioxidative stress’ (58). Giving a single agent might supply only one spot of the big networking framework that eventually did not lead to the overall beneficial outcomes. The synergistic effect of a wide array of phytoantioxidants would rather help orchestrate the antioxidant defense as a whole. Thus, it is important to provide a wide range of phytochemicals to create an effective milieu of ROS-scavenging defense. Daily consumption of a variety of tropical fruits is warranted for the prevention of CVD.

## Conclusions

Emerging trends in the food industry have been shaped by functional and novel food, just as well as nanotechnology. High intakes of functional food, such as fruits and vegetables, reportedly promote health and, under certain circumstances, prevent diseases. All 10 fruit samples possess antioxidant activities but only AS and CP-u exhibit functional activities to protect endothelial cells against oxidative damage and promote endothelial wound healing. This study encourages the consumption of a wide variety of tropical fruits for health benefits, especially CVD protection, and highlights the importance of further food product development for health-concerned consumers. Nevertheless, further studies involving human patients should be validated prior to claiming the disease preventive effect of functional foods.
